# Association between baseline abundance of *Peptoniphilus*, a Gram-positive anaerobic coccus, and wound healing outcomes of DFUs

**DOI:** 10.1371/journal.pone.0227006

**Published:** 2020-01-24

**Authors:** Kyung R. Min, Adriana Galvis, Katherine L. Baquerizo Nole, Rohita Sinha, Jennifer Clarke, Robert S. Kirsner, Dragana Ajdic

**Affiliations:** 1 Dr. Phillip Frost Department of Dermatology & Cutaneous Surgery, University of Miami, Miller School of Medicine, Miami, Florida, United States of America; 2 Department of Statistics, University of Nebraska-Lincoln, Lincoln, Nebraska, United States of America; University of Illinois College of Medicine, UNITED STATES

## Abstract

Diabetic foot ulcers (DFUs) lead to nearly 100,000 lower limb amputations annually in the United States. DFUs are colonized by complex microbial communities, and infection is one of the most common reasons for diabetes-related hospitalizations and amputations. In this study, we examined how DFU microbiomes respond to initial sharp debridement and offloading and how the initial composition associates with 4 week healing outcomes. We employed 16S rRNA next generation sequencing to perform microbial profiling on 50 samples collected from 10 patients with vascularized neuropathic DFUs. Debrided wound samples were obtained at initial visit and after one week from two DFU locations, wound bed and wound edge. Samples of the foot skin outside of the wounds were also collected for comparison. We showed that DFU wound beds are colonized by a greater number of distinct bacterial phylotypes compared to the wound edge or skin outside the wound. However, no significant microbiome diversity changes occurred at the wound sites after one week of standard care. Finally, increased initial abundance of Gram-positive anaerobic cocci (GPAC), especially *Peptoniphilus* (p < 0.05; n = 5 subjects), was associated with impaired healing; thus, GPAC’s abundance could be a predictor of the wound-healing outcome.

## Introduction

Diabetes mellitus (DM) affects over 422 million people worldwide and nearly 30 million Americans [1–3]. Diabetic foot ulcers (DFUs) are chronic wounds that develop upon skin injury in patients with long-standing and often poorly-controlled DM. DFUs occur in up to 25% of all patients with DM [4, 5], and even with standard care, one in three DFUs fails to heal [1, 2, 6, 7] preceding 85% of all diabetes-related lower-leg amputations [8]. DFU patients are highly susceptible to rapidly spreading infection that can lead to soft tissue damage and osteomyelitis, lower limb amputation (approximately 100,000 cases annually in the USA), sepsis, and death [1, 2, 6, 7].

DFUs are colonized by microorganisms, and bacteria are routinely isolated from DFUs by standard culturing methods. However, these methods fail to identify many bacterial species colonizing DFUs because these species grow poorly or cannot be cultured in the laboratory [9]. High-throughput DNA sequencing methods permit a culture-independent examination of the bacterial composition with extraordinary depth [10, 11]. In fact, 16S rRNA sequencing-based microbial profiling detects many more complex wound microbiota than described previously using culture-based analyses.[12, 13] Multiple DFU microbiome studies have reported diverse microbiota in DFUs with *Staphylococcus*, *Streptococcus*, *Corynebacterium*, and many obligate anaerobes being the most prevalent [12–14]. Additionally, both pathogenic and commensal bacterial species were observed in DFUs [15]. A well-designed study by Gardner *et al*. demonstrated an association between DFU microbiomes and clinical factors such as ulcer depth, duration, and poor glycemic control [14]. However, it wasn’t until recently that a longitudinal study of DFU’s microbiomes described the temporal dynamics of microbial composition throughout the course of DFU management [16]. The most recent data from metagenomic sequencing shows that specific strains of *S*. *aureus* are associated with impaired wound healing, suggesting the importance of *S*. *aureus* strain variations in clinical outcomes [17].

Currently, the standard care for DFUs includes sharp debridement and offloading using a removable boot or, occasionally, a contact cast to relieve pressure in the affected foot [5, 18]. Even with quality standard care, more than 30% of patients with DFUs fail to heal, resulting in lower limb amputations [5, 19, 20]. Furthermore, antibiotic therapy has not shown to improve healing of non-infected DFUs [21–23]. Debridement provides clinical benefits through the removal of unresponsive and necrotic tissue [5, 24]. This process is repeated weekly to stimulate wound repair and enhance healing.[25] Current research is beginning to examine the temporal dynamics of wound microbiota [16, 17, 26, 27]; however, the extent to which initial debridement affects the DFU microbiota is still unclear.

In this study, we addressed if one week of standard care alters DFU microbiome composition and if the initial composition associates with 4 week healing outcomes. We collected debrided specimens from 10 patients with vascularized neuropathic DFUs at initial visit and at a follow-up one week later, and performed 16S rRNA microbiome profiling using next generation sequencing. We compared the changes in the diversity, abundance, and taxonomic composition of DFU microbiota following one week of standard care. Finally, we addressed the hypothesis that there might be an association between healing outcome and the DFU microbiome’s initial composition.

## Materials and methods

### Patients and samples

The patient demographics and clinical parameters are presented in Table 1 and S1 Table. This study was approved by the University of Miami Miller School of Medicine Institutional Review Board (CR00001542). Specimens of human skin were obtained from patients with DM and plantar DFU. A total of 10 patients were enrolled and provided standard DFU treatment for 4 weeks which included baseline wound debridement and offloading. Wound size was measured weekly for 4 weeks and healing, as measured by percentage of wound-size reduction, was assessed after 4 weeks of standard care.

**Table 1:** Patient demographics and clinical parameters.

Parameters	Healing Wounds	Non-Healing Wounds
Patients	5	5
Sex	2 Males, 3 Females	3 Males, 2 Females
Median Age	73	56
Diabetes Type	All type II	3 type II, 2 type I
Median Ulcer Duration (months)	4	8
Antibiotic therapy	None	None
HbA1c (± SD)	7.3 ± 1.3	7.4 ± 1.6
ABI (± SD)	1.114 ± 0.201	1.092 ± 0.074

Inclusion criteria included the following: 21 years of age or older; vascularized plantar neuropathic DFU with duration of at least 4 weeks and larger than 0.5 cm^2^; and compliance with the use of off-loading diabetic boot. Subjects were excluded from the study for having any of the following criteria: use of topical or systemic antibiotics over the course of 2 weeks prior to debridement; active cellulitis or osteomyelitis; vascular insufficiency; ankle–brachial index lower than 0.7 or higher than 1.3; revascularization to the ipsilateral lower extremity in the last 6 weeks; history of immunodeficiency or neoplastic lesions in ulcer area; or use of experimental treatments or drugs in the last 4 weeks. The level of glycemic control was measured as hemoglobin HbA1c values [28, 29].

There were equal numbers of men and women enrolled (Table 1). Eight patients had type-2 DM and 2 type-1 DM. Hemoglobin A1c for the patients were between 5.7 and 8.7, except for one patient (10.1). Patients received standard care with off-loading removable diabetic boot for the entire study, removed only at weekly study visits. During the first and second study visits, ulcer tissue samples and swabs of the foot skin outside the wound were collected. The ulcers were then covered with foam dressing. Ulcer size measurements and photographs were collected weekly for 4 weeks. Wound duration was measured as the number of weeks from the time of soft tissue loss to the baseline visit obtained through subject report and review of medical records.

### Ulcer healing

As a surrogate endpoint for complete healing, we used the well-established 50% wound size reduction after 4 weeks [30, 31]. Wounds that were ≥ 50% closed by week 4 were defined as “healing wounds”; all other wounds were defined as “non-healing wounds”. Ulcer size, including surface area and depth, was measured using planimetry. Ulcer size measurements were done pre- and post-debridement weekly for the duration of the study.

### Sample collection

Skin swabs were collected before cleansing from the medial, plantar part of the foot, 2.5 cm away from the wound dressing. Skin swabs were obtained using the Levine technique [32] weekly in the first 2 weeks of the study. The swab was moistened in sterile PBS and rotated over a 1 cm^2^ area of the foot skin for 5 seconds using sufficient pressure. Swabs were taken from the foot skin without occlusion. A total of 20 skin swabs were collected.

Ulcer specimens were obtained before cleansing by debridement of the wound edge (skin immediately adjacent to the wound bed) using a scalpel and debridement of the entire wound bed with a curette at patients’ initial visit and at a follow-up visit one week later. A total of 20 wound-bed and 20 wound-edge samples were collected. Special care was taken not to cross-contaminate samples during the collection.

All specimens were submerged in PBS-based collection fluid (MoBio), placed at -80°C within one hour from collection and stored until DNA isolation.

Afterwards, ulcers that needed debridement underwent further debridement without tissue collection. Hemostasis was achieved with local pressure or, only if necessary, with topical hemostatic agents (Aluminum chloride 20%) or electrocautery at the discretion of the surgeon.

### DNA isolation

DNA was isolated from tissue samples with the ZR Fungal/Bacterial DNA Mini Prep^™^ kit (Zymo Research, Irvine, CA) according to manufacturer’s protocol and optimized for high-throughput processing. Cell lysis was carried out with approximately 30–40 mg of DFU tissue samples using 1 ml of sterile zirconia beads containing an equal mixture of 0.1 mm and 0.5 mm beads. Bead-beating was carried out in a FastPrep-24^™^ homogenizer (MP Biomedicals, Santa Ana, CA) at 4.5 m/s for 60 seconds with 1 min cooling on ice in between for a total number of 4 cycles. All samples were quantified via the Qubit^®^ / Quant-iT dsDNA High Sensitivity Kit (Invitrogen, Life Technologies, Carlsbad, CA). A total of 60 samples were processed (S1 Table). Twenty wound bed, twenty wound edge, and ten adjacent skin samples yielded sufficient 16S rDNA for sequencing. Sample collection controls were also processed but 16S rDNA was not detected in the controls; thus, they were not sequenced.

### Library preparation and sequencing

Library preparation and sequencing was carried out by Second Genome, Inc. (San Francisco, CA). To enrich the samples for bacterial V4 region of the 16S RNA gene, DNA was PCR amplified in 25 μl reactions using 10 ng of template DNA with 5Prime HotMasterMix (Quantabio, Beverly, MA) and 5 pmol of primers 515F (5’-GTG CCA GCM GCC GCG GTA A-3’) and 806R (5’-GGA CTA CHV GGG TWT CTA AT-3’). Thermocycling consisted of an initial denaturation step at 94°C for 3 min, followed by 25 cycles of denaturation at 94°C for 45 sec, annealing at 50°C for 60 sec, and extension at 72°C for 90 sec, and a final extension step at 72°C for 10 min. The barcodes were attached to the indexed reverse primers. Following amplification of bacterial V4 target sequences, PCR products were purified using the Agencourt AMPure XP clean-up kit according to manufacturer’s protocol (Beckman Coulter, Brea, CA). Amplicons were then quantified using the Quant-iT PicoGreen dsDNA Assay Kit as per manufacturer’s instructions (Thermo-Fisher Scientific, Waltham, MA). Equimolar amounts of all sample amplicons were pooled into a single multiplexed library and the quality and molarity of the resulting library were assessed using a BioAnalyzer High Sensitivity DNA chip (Agilent Technologies, Santa Clara, CA). Barcoded samples were loaded into a MiSeq^®^ reagent cartridge, and then onto the instrument (Illumina MiSeq). The samples were sequenced for 250 cycles with custom primers designed for paired-end sequencing. Negative controls for the library preparations and sequencing included samples in which molecular grade water was used instead of DNA template. A known mixture of “mock” microbiome sample was included as a positive control.

The sequencing data has been deposited to the NCBI Sequence Read Archive database (accession number PRJNA579196).

### OTU selection

Sequenced paired-end reads were merged, quality filtered, and de-replicated with USEARCH [33]. Resulting unique sequences were then clustered at 97% similarity by UPARSE (de novo OTU clustering) and a representative consensus sequence per de novo OTU was determined. The clustering algorithm also performed filtering to discard likely chimeric OTUs. Sequences that passed quality filtering were then mapped to a set of representative consensus sequences to generate an OTU abundance table. Representative OTU sequences were assigned taxonomic classification via mothur’s Bayesian classifier at 80% confidence; the classifier was trained against the Greengenes reference database [34] of 16S rRNA gene sequences clustered at 99%. After the taxa were identified for inclusion in the analysis, the values used for each taxa-sample intersection were populated with the abundance of reads assigned to each OTU. The data was normalized by calculating relative OTU abundance (S2 Table).

### Data analysis and statistical tests

Bacterial diversity and community analyses were performed using the R statistical computing language [35] ver 3.4.3 and the package vegan [36] ver 2.4.6. Alpha-diversity metrics were assessed using the functions *specnumber* for sample richness determination and *diversity* for Shannon-Weaver diversity indices. Pielou’s evenness was calculated as a ratio of the Shannon diversity index over the log of richness. For calculations of the alpha diversity metrics, we used non-rarified relative abundance values. Statistical significance testing was carried out using a one-way analysis of variance (ANOVA) followed by post-hoc analysis using Tukey’s honest significant difference test (HSD).

Beta-diversity metrics were assessed using the vegan function *metaMDS* to generate a community dissimilarity distance matrix for ordination using a non-metric multidimensional scaling approach. For significance testing of factors and interactions that affect bacterial compositions, a permutational multivariate analysis (PERMANOVA) was carried out using the function *adonis* on the *Bray-Curtis* dissimilarity matrix.

For statistical significance testing of taxonomic composition, analysis of composition of microbiomes (ANCOM)[37] was performed using the R package ancom.R ver 1.1.3 and scikit-bio 0.5.0. Only the wound bed and wound edge samples were analyzed for significance testing of the relative abundance at the phylum and genus levels.

Power analysis was performed using the R package micropower [38].

## Results

### Microbiome profiling of DFUs

To investigate how the initial debridement influences the composition of wound microbiota, we profiled the microbiome of DFU samples collected from patients at initial visit and at follow-up after one week. Specifically, tissue samples were collected by debridement from wound edge and wound bed, and swabs from the foot skin outside the wound. Total DNA was isolated from all samples (n = 60). However, we could not detect any DNA in 10 skin-swab samples (S1 Table) and these samples were not sequenced.

Microbial profiling was performed using the V4 conserved regions of bacterial 16S rRNA with Illumina MiSeq Next Generation Sequencing. A summary of the sequencing information is presented in Table 2. Sequencing resulted in excess of 16 million total raw reads. Merging of paired reads and quality filtering resulted in ~6.4 million reads. More then 6.1 million reads were assigned taxonomic identification. After de-replication, clustering and taxonomic assignment, we detected 1,478 total *de novo* operational taxonomic units (OTUs). We performed independent filtering to remove OTUs that were spurious (i.e. OTUs that were not seen at least once within 10% of the dataset) which resulted in 563 OTUs. We also removed contaminating OTUs that were found in the negative controls for library preparation. These filtering processes decreased the OTU number to 525 and the resulting OTUs were used in all subsequent analyses.

**Table 2:** Sequencing summary.

Total reads	16,389,328
Total reads paired	8,107,765
Total filtered reads	6,412,740
Total reads matched to raw OTUs	6,131,576
Total reads matched to filtered OTUs	6,131,200
Library size (Mean ± SD)	145,987 ± 60,617
Average read length	251
Average length of paired reads	252
Total de novo raw OTUs	1,577
Total de novo filtered OTUs	1,478
Total OTUs after independent filtering	563
Total OTUs after removal of contaminating OTUs	525

The sample library size was normally distributed around the mean and standard deviation of 145,987 ± 60,617 (Table 2). We did not observe any significant differences in sample library sizes due to the different sample collection techniques (tissue debridement versus swabs, t-test *p* = 0.459).

### Impact of clinical covariates on DFU microbiota

The significance of clinical covariates on DFU wound bed and wound edge microbiota were assessed using PERMANOVA, and their impact on beta-diversity using the Bray-Curtis dissimilarity is presented in a NMDS ordination (stress = 0.21) (S1 Fig). Analysis of clinical covariates yielded statistical significance for individual subject (`Patient ID’), sampling location (‘Location’), healing outcome (‘Outcome’), Diabetes Type (‘Diabetes’), HbA1C, ABI, Gender, Age, and wound duration (‘Duration’) (S1 Fig). Among these, HbA1C, Gender and Patient ID contributed to the greatest difference in microbiota. Given the limited number of subjects enrolled in this study, the individual subject microbiota differences appear to underlie these confounding clinical covariates.

No statistically significant correlations could be made between the metadata and wound healing outcome.

### Bacterial diversity in DFUs

**Fig 1:**
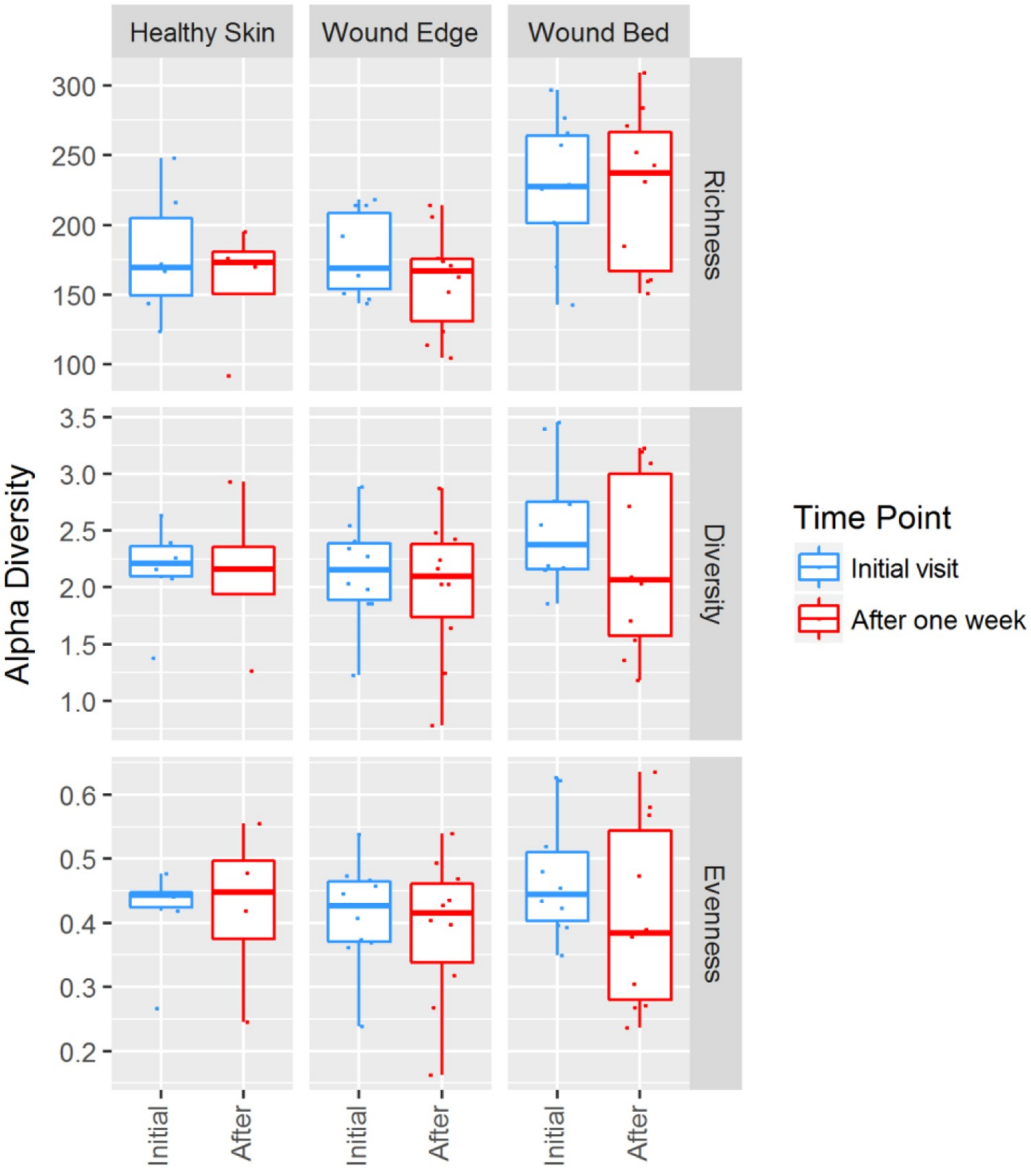
Bacterial diversity based on sampling location and time point. The alpha diversity indices showing sample richness (richness), Shannon-Weaver diversity (diversity) and Pielou’s evenness (evenness) are presented based on sampling location and time point. Healthy Skin–swabs outside of wound area, Wound Bed–debrided tissue from a wound base, Wound Edge–debrided peripheral tissue surrounding a wound.

We compared the alpha diversity indices such as richness, Shannon-Weaver diversity, and Pielou’s evenness to evaluate the differences in the bacterial diversity of DFUs. The sample richness represents the number of unique taxa found regardless of abundance. The Shannon-Weaver diversity index, on the other hand, represents a measure of the distribution of all taxa based on their abundance. A higher Shannon-Weaver index indicates the presence of a greater number of substantial taxa. Pielou’s evenness provides a measure of dominance in a community with a maximum value of 1 representing equal abundance by all taxa and a minimum value of 0 representing dominance by a single taxon. The results of alpha diversity analysis are presented in Fig 1 based on 1) sampling location and 2) time of patient visit. Overall, we found that the wound bed samples were colonized by a significantly greater number of mean unique taxa (204) compared to foot skin (149) or wound edge (150) samples (ANOVA F(2,47) = 9.583, *p* < 0.001). Taking into consideration the relative abundance of each taxon, no significant differences in Shannon-Weaver diversity (*p* = 0.281) or Pielou’s evenness (*p* = 0.647) were observed. When we compared the bacterial diversity in DFUs at initial visit and one week later, no significant differences were observed in the bacterial alpha diversity indices (2-Way ANOVA Location x Time *p* > 0.7) (Fig 1).

### Analysis of dissimilarities in the composition of DFU microbiota

**Fig 2:**
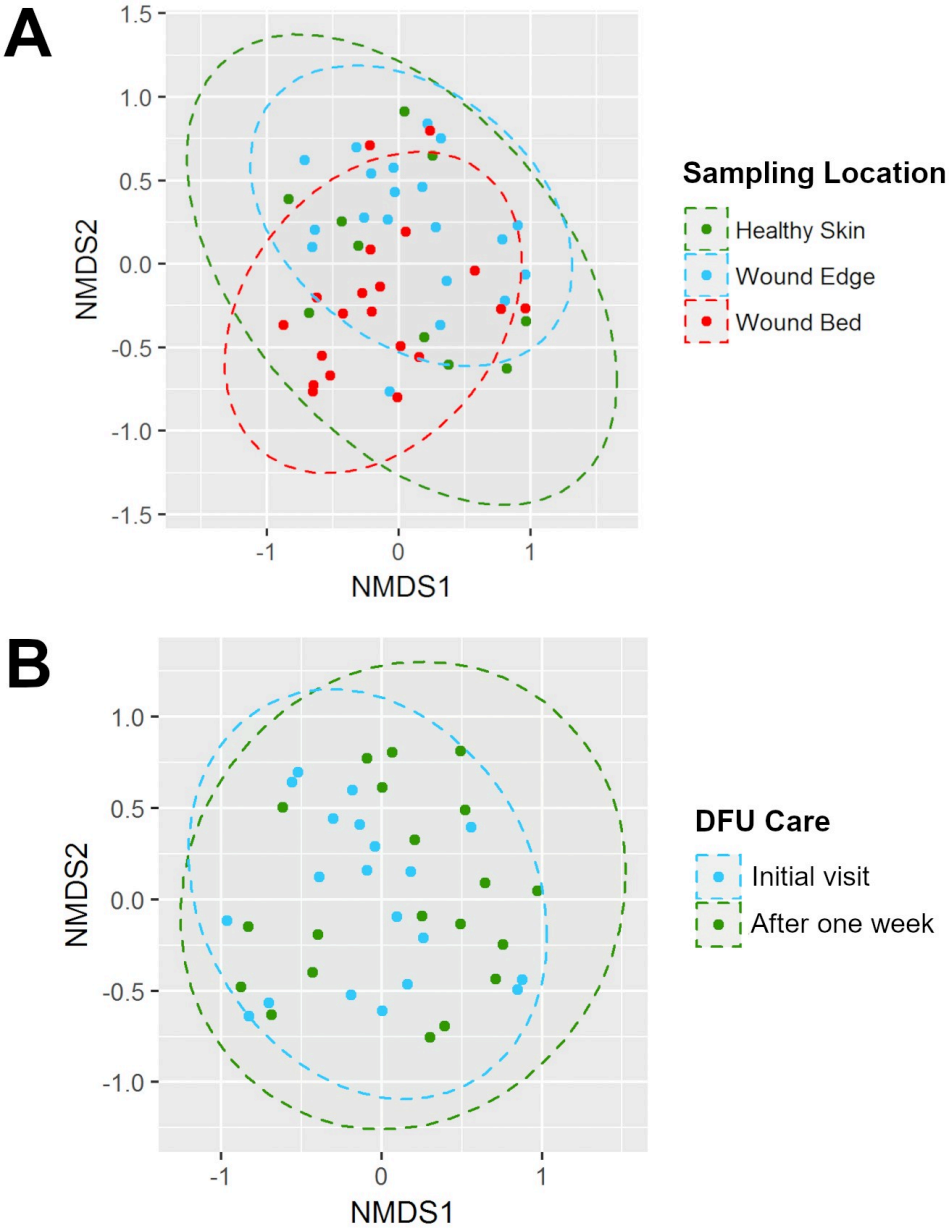
Bray-Curtis based ordination of DFU microbiomes (β-diversity). Non-metric multidimensional scaling (NMDS) plot (goodness of fit or stress = 0.21) showing the compositional dissimilarities of all samples collected in this study according to (A) sampling location and (B) time point (DFU care). Ellipsoids represent a 95% confidence interval used to group each sample.

**Fig 3:**
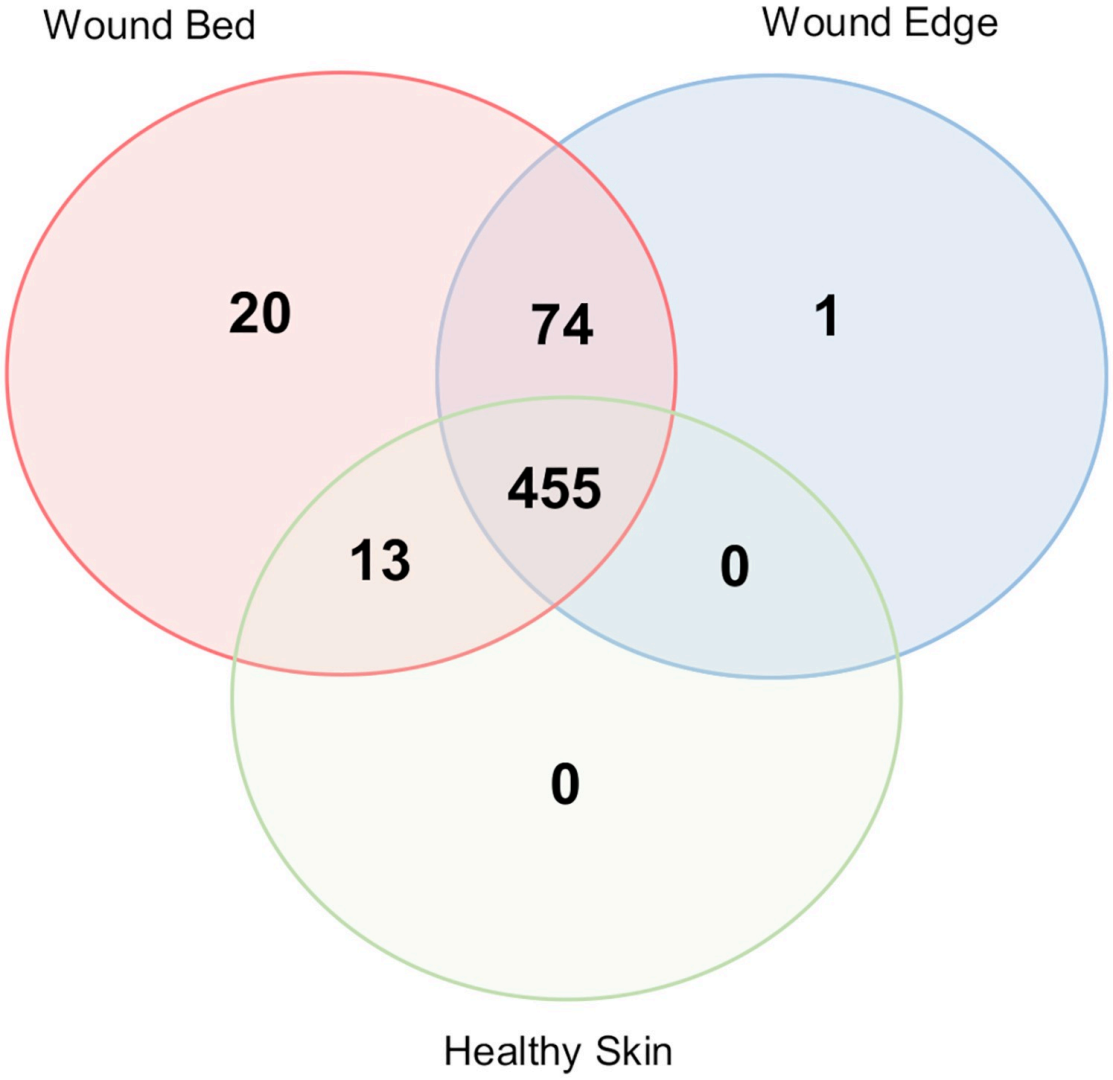
Community membership analysis. A 3-way Venn diagram showing unique and shared taxa between samples from different locations. Selection criteria was based on presence or absence regardless of abundance. Not drawn to scale.

To determine the differences in the composition of DFU microbiome between wound bed, wound edge, and foot skin, at initial visit and one week after administration of standard DFU care, we performed beta diversity analysis by calculating a pair-wise dissimilarity matrix of the relative abundance for all OTUs in the samples collected using the Bray-Curtis dissimilarity. This index quantifies the differences in both, composition and abundance of biological communities along a gradient or across distinct sites with a maximum value of 1 representing no shared taxa and a minimum value of 0 representing total similarity. The resulting matrix dissimilarities were rank ordered and ordination was carried out using the non-metric multidimensional scaling. The results presented in Fig 2 showed the distribution of each sample’s compositional dissimilarity across two non-metric dimensions. The more dissimilar the composition is between the samples, the further they are located relative to one another. The results showed no distinct separation of wound microbiota based on sampling location (Fig 2A) or one week of standard DFUs’ care (Fig 2B). All the DFU sample clusters overlapped with one another and indicated that their bacterial compositions shared a large degree of similarity. To address the extent to which these DFU microbiota differed, we performed a follow-up community membership analysis to determine the distribution of unique OTUs across the different sampling locations regardless of abundance. This was carried out using the presence and absence criteria as described by Shade and Handelsman [39]. The result of this analysis is presented as a Venn diagram in Fig 3 and corroborates the findings of the ordination in Fig 2A. Among all OTUs analyzed in this study (525), we found that almost 80% (419) of OTUs were shared between the DFU samples. Twenty OTUs were only present in wound bed samples, 73 were shared between wound bed and wound edge samples, and 12 were shared between wound bed and foot skin. No unique OTUs were found in the foot skin samples.

### Taxonomic composition of DFU microbiome

**Fig 4:**
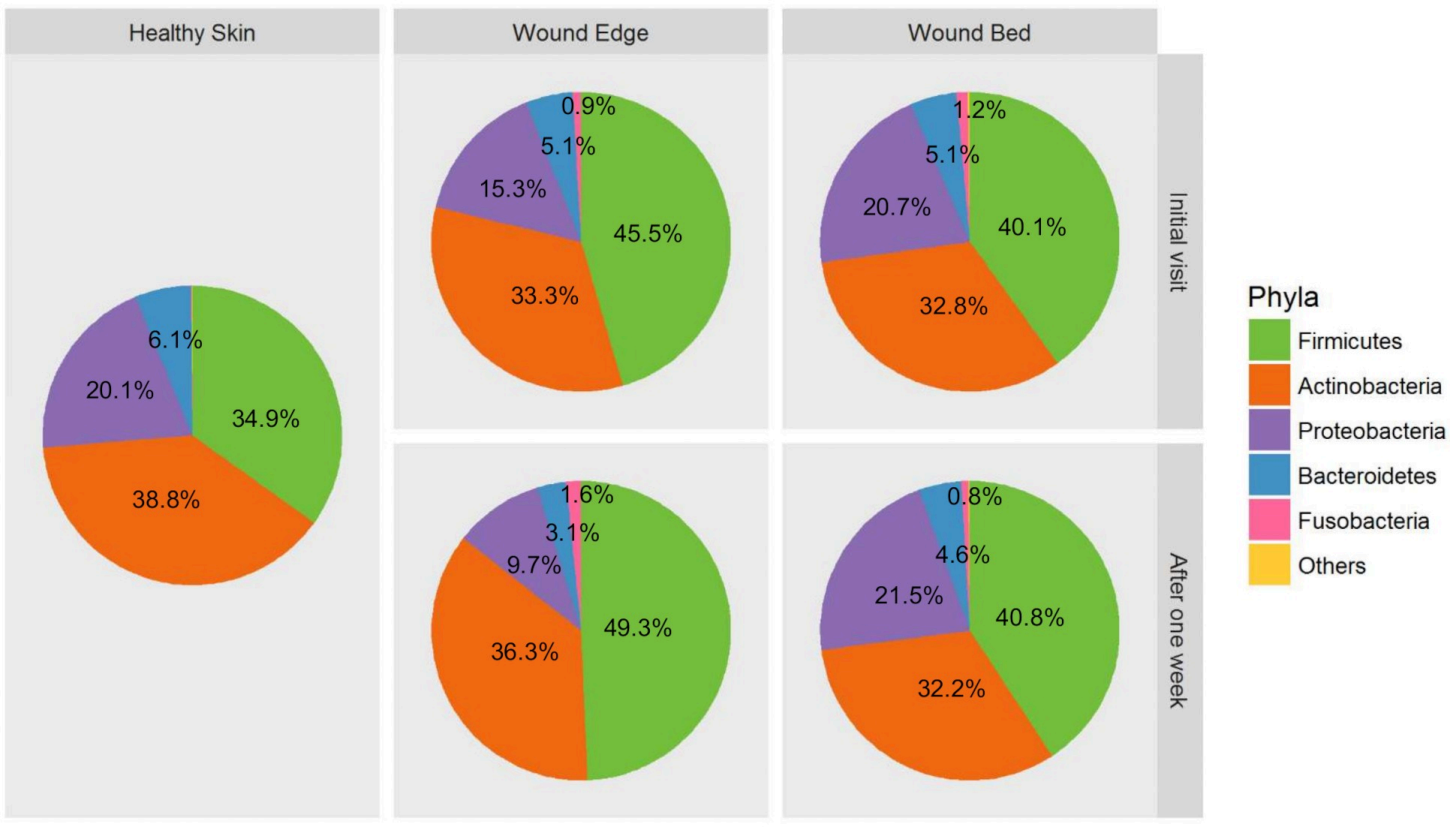
Microbiome composition of DFU samples. Pie charts showing the relative abundance and composition of the most dominant phyla according to sampling location and time point.

**Fig 5:**
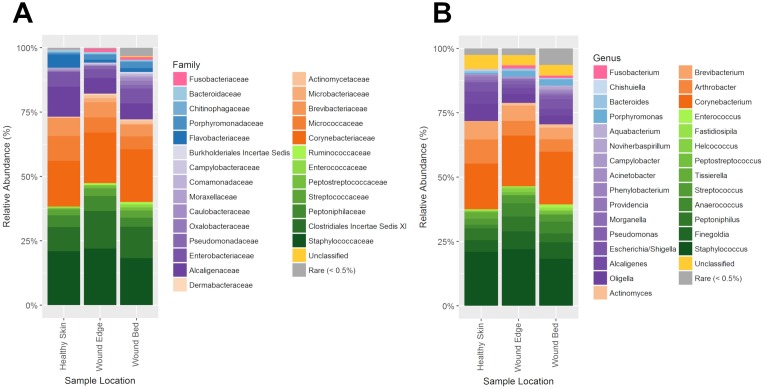
Mean relative abundance of bacterial families and genera in DFU microbiomes. Stacked bar plots showing the composition of the most dominant (A) families and (B) genera color-coded in order of phylum and ranked according to abundance. A relative abundance greater than 0.5% was considered abundant in this analysis. Pink, *Fusobacteria*; Blue, *Bacteroidetes*; Purple: *Proteobacteria*; Orange, *Actinobacteria*; Green, *Firmicutes*.

Analysis of taxonomic composition revealed that the microbiota of DFUs comprise an average of 172 ± 50 phylotypes per sample based on presence/absence. However, an average of 14 ± 6 OTUs among these had a relative abundance greater than 0.5% and indicated that only approximately 8% of all OTUs make up the bulk of the microbial communities while the rest are present in fractional abundance. When we examined the taxonomic identities of OTUs, their classifications span across 13 different phyla, 92 families, and 142 genera (Table 3). An assessment of the mean relative abundance in collected samples provided an overview of the dominant taxa (Fig 4). On average, the microbiome of foot skin and DFUs comprised of five dominant phyla that included *Firmicutes* (44.7%), *Actinobacteria* (35.1%), *Proteobacteria* (14.5%), *Bacteroidetes* (4.6%) and *Fusobacteria* (0.9%). The remaining 8 phyla were not abundant and collectively comprised less than 0.2% of all OTUs. Some differences in the taxonomic composition at the phylum level were observed for the different sampling locations (Fig 4). The relative abundance of *Fusobacteria* (pink) was negligible in foot skin; *Firmicutes* (green) were more abundant in wound edge tissue samples; and *Proteobacteria* (purple) were more abundant in wound bed tissue samples; however, these differences were not statistically significant (*p* > 0.05). When we examined the microbial composition at the phylum level in wound edge and wound bed tissue samples collected at initial visit versus one week later, we did not observe statistically significant changes (*p* > 0.05). At lower taxonomic ranks, further assessment of the mean relative abundance revealed that of the 92 families present in DFU samples, 26 were dominant (Fig 5A) while 28 out of 142 genera were dominant (Fig 5B). The most abundant families included *Staphylococcaceae* (21.1%), *Corynebacteriaceae* (20.2%), *Clostridiales Incertae Sedis XI* (12.5%), *Alcaligenaeceae* (8.3%), *Micrococcaceae* (7.1%), *Brevibacteriaceae* (6.0%), and *Peptoniphilaceae* (4.7%). Substantial genera included *Staphylococcus* (21.1%), *Corynebacterium* (20.3%), *Arthrobacter* (6.9%), *Finegoldia* (6.1%), *Brevibacterium* (6.0%), *Peptoniphilus* (4.7%), *Oligella* (4.7%), *Anaerococcus* (3.9%), *Alcaligenes* (3.4%), *Streptococcus* (2.7%), *Porphyromonas* (1.7%) and *Pseudomonas* (1.0%).

**Table 3:** Unique identified taxa.

Rank	Number
Kingdom	1
Phylum	13
Class	28
Order	46
Family	92
Genus	142

### Impact of the initial DFU microbiome’s composition on healing outcome

**Fig 6:**
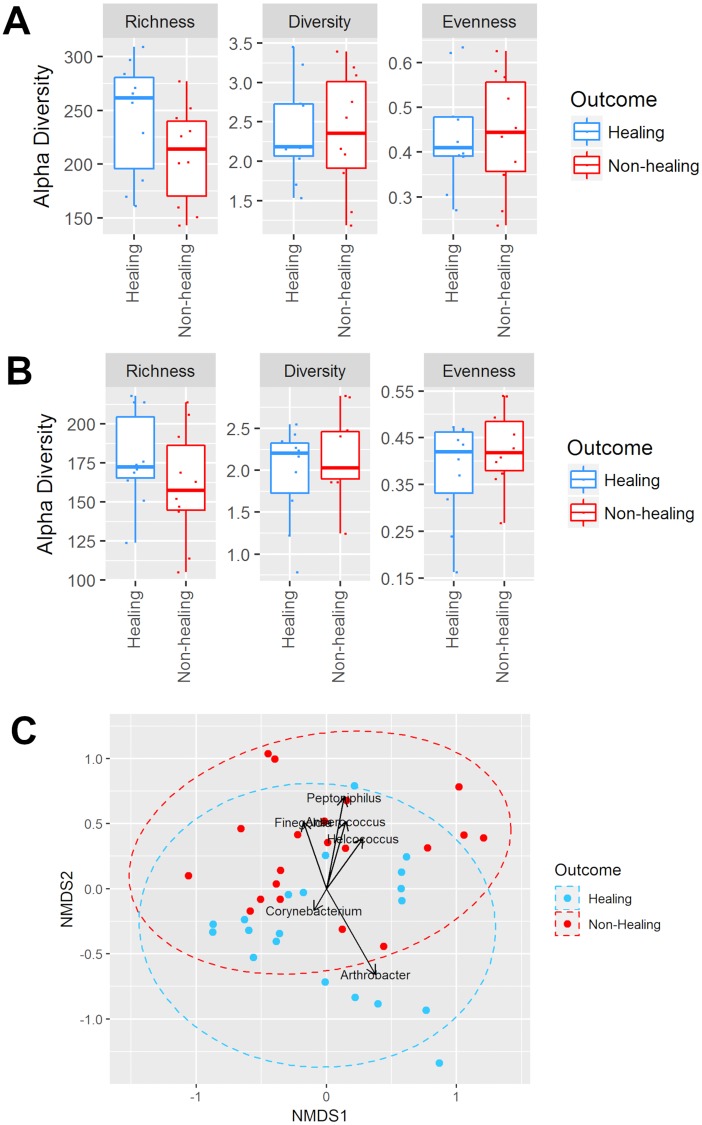
Bacterial diversity in healing and non-healing DFU microbiomes. Diversity of the DFU microbiomes was compared in the samples collected at the initial visit, grouping the samples based on the healing outcome. The alpha diversity indices showing sample richness (richness), Shannon-Weaver diversity (diversity) and Pielou’s evenness (evenness) are presented based on healing outcome for (A) wound bed and (B) wound edge samples. (C) The compositional dissimilarity in the microbiome of healing and non-healing wounds is presented as a non-metric multidimensional scaling (NMDS) plot (goodness of fit or stress = 0.21) based on the ordination of Bray-Curtis dissimilarity (β-diversity). Direction and length of arrows indicate which GPAC contributed to the microbiota compositions of wounds that healed (*Arthrobacter and Corynebacterium*) or did not heal (*Peptoniphilus*, *Anaerococcus*, *Finegoldia*, and *Helcococcus*).

We compared alpha diversity of the DFU microbiomes in the samples collected at the initial visit, grouping the samples based on the assessment of healing outcome. For the wound bed samples, the results showed no statistically significant differences in sample richness, Shannon-Weaver diversity, or community evenness (*p* > 0.05, Fig 6A). Similar results were observed for wound edge samples (p > 0.05, Fig 6B). Analysis of compositional dissimilarities based on the ordination of Bray-Curtis also showed no distinct separation of DFU samples (Fig 6C), indicating that the microbiota composition of DFUs that healed compared to those that did not heal shared a large degree of similarity.

**Fig 7:**
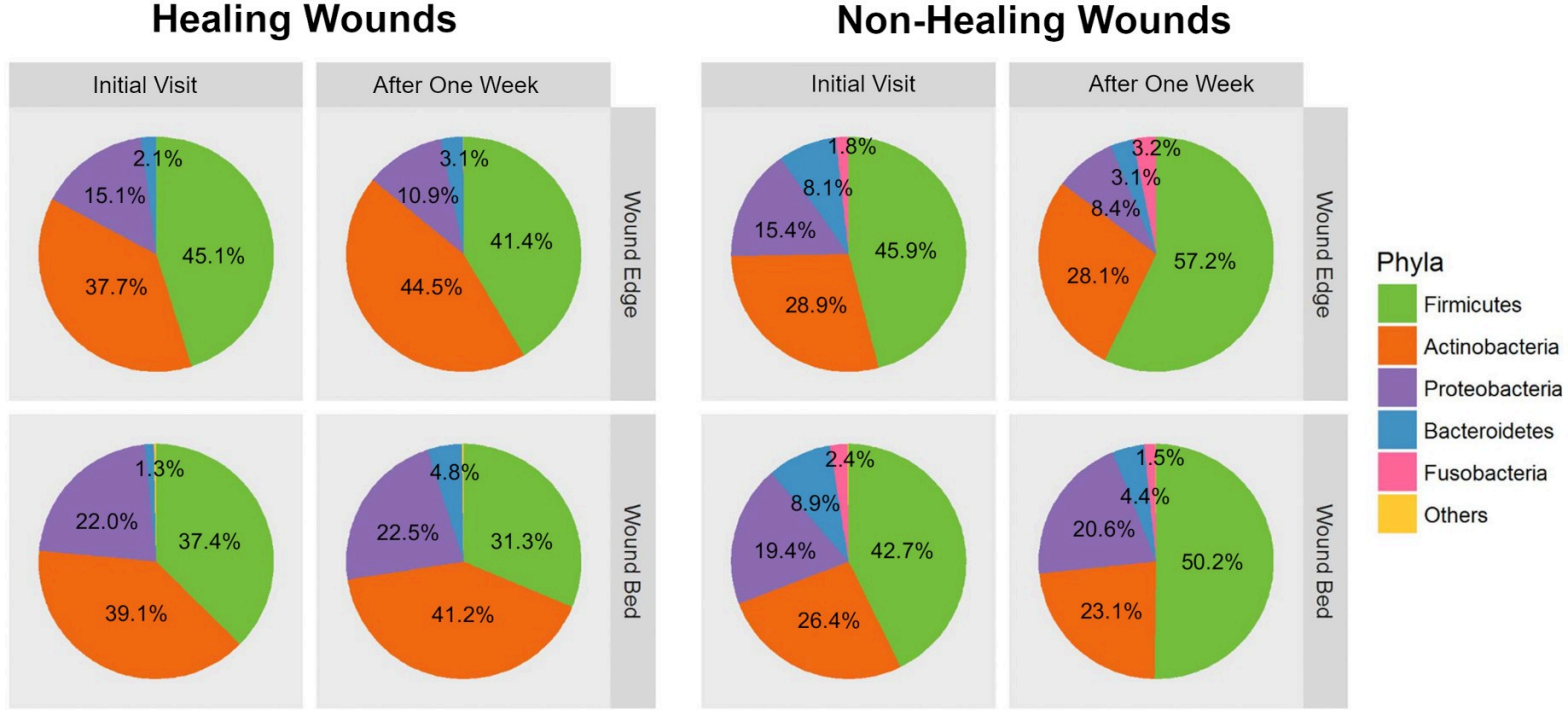
Impact of initial DFU microbiome composition on healing outcome. Pie charts showing the distribution of the most dominant phyla according to healing outcome, sampling location, and time point.

**Fig 8:**
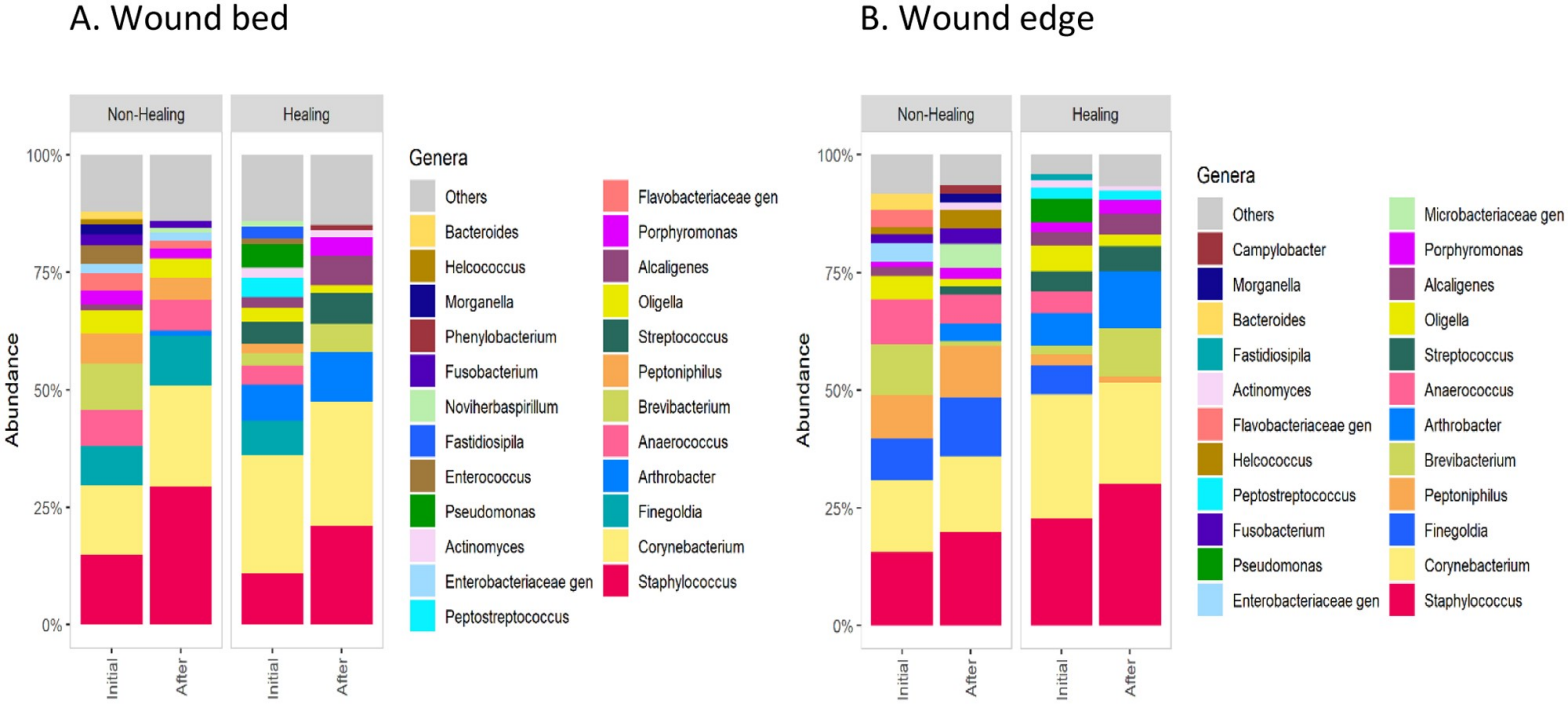
Stacked bar plots showing the composition of predominant genera in healing and non-healing (A) wound bed or (B) wound edge samples before and after standard care. A relative abundance greater than 0.5% was considered in this analysis.

We also examined the distribution of microbial abundance at the phylum level in wound bed and wound edge samples taken at both time points. In initial samples from DFUs that later showed impaired healing, we observed an increased abundance of *Firmicutes*, *Bacteroidetes*, and *Fusobacteria*, and a reduction of *Actinobacteria* compared to the healing DFUs (Fig 7); however, these differences were not statistically significant (p > 0.05). In contrast, we observed statistically significant abundance increase of the genus *Peptoniphilus* (p < 0.05) based on the frequency of occurrence and mean abundance of dominant taxa (≥ 5%) in the wound bed and wound edge samples of the DFUs with impaired healing (n = 5 subjects; n = 20 samples per group) (Table 4, Figs 6C and 8). We also observed an increase in abundance of *Finegoldia*, *Anaerococcus*, and *Helcococcus* in the same samples (Table 4, Figs 6C and 8). Although this increase was not statistically significant (p > 0.05), we had 70% power to detect significant differences for *Finegoldia and Anaerococcus* in our samples (n = 5 subjects; n = 20 samples per group).

**Table 4:** Mean relative abundance of dominant taxa (≥ 5%) in wound samples of healing and non-healing DFUs.

Genera / Species	Phylum	Healing (n = 20)		Non-Healing (n = 20)	
		n	Mean Abundance (%)	n	Mean Abundance (%)
*Staphylococcus*	Firmicutes	12	34.46 (± 24.33)	11	34.58 (± 21.29)
*Finegoldia magna*	Firmicutes	2	29.00 (± 4.26)	14	13.55 (± 6.90)
*Anaerococcus*	Firmicutes	5	7.31 (± 2.92)	7	13.31 (± 9.17)
*Peptoniphilus gorbachii*	Firmicutes	2	6.37 (± 0.04)	10	8.90 (± 4.38)
*Enterococcus*	Firmicutes	1	6.12	1	17.59
*Streptococcus agalactiae*	Firmicutes	6	16.53 (± 9.42)	-	-
*Peptostreptococcus anaerobius*	Firmicutes	3	13.94 (± 5.28)	-	-
*Helcococcus*	Firmicutes	-	-	2	7.60 (± 1.95)
*Helcococcus kunzii*	Firmicutes	-	-	1	10.41
*Corynebacterium*	Actinobacteria	19	21.93 (± 12.03)	14	22.13 (± 10.20)
*Arthrobacter cumminsii*	Actinobacteria	4	36.66 (± 9.07)	1	12.09
*Arthrobacter*	Actinobacteria	4	8.53 (± 1.97)	1	5.31
*Brevibacterium luteolum*	Actinobacteria	2	17.42 (± 9.56)	2	44.19 (± 0.56)
*Actinomyces europaeus*	Actinobacteria	2	6.12 (± 1.58)	1	5.1
*Brevibacterium*	Actinobacteria	4	11.95 (± 3.73)	-	-
*Corynebacterium aurimucosum*	Actinobacteria	2	17.66 (± 1.42)	-	-
*Corynebacterium tuberculostearicum*	Actinobacteria	1	17.25	-	-
*Actinomyces neuii*	Actinobacteria	1	7.14	-	-
*Oligella urethralis*	Proteobacteria	4	14.13 (± 8.77)	4	18.60 (± 7.34)
*Alcaligenes spp*.	Proteobacteria	5	14.71 (± 8.13)	1	7.34
*Pseudomonas aeruginosa*	Proteobacteria	2	21.58 (± 1.37)	-	-
*Morganella morganii*	Proteobacteria	-	-	2	10.26 (± 0.06)
*Campylobacter*	Proteobacteria	-	-	1	8.54
*Porphyromonas*	Bacteroidetes	3	14.84 (± 4.67)	4	8.02 (± 2.46)
*Bacteroides fragilis*	Bacteroidetes	-	-	2	11.97 (± 6.09)
*Fusobacterium*	Fusobacteria	-	-	4	11.19 (± 3.76)

Additionally, we have performed a power analysis looking at comparisons between healing and non-healing wounds for initial wound edge or wound bed samples. We assume, based on data reported here, that 3% of OTUs are unique to either group and the average number of reads per OTU is approximately 250. We would need 30 subjects per group to have at least 80% (initial wound edge) power or 70% (initial wound bed) power to detect any significant difference at the OTU level between groups at adjusted p<0.05.

The increased abundance of *Peptoniphilus*, *Finegoldia*, *Anaerococcus*, and *Helcococcus*, all classified as Gram-positive anaerobic cocci (GPAC), contributed to the increase of *Firmicutes* in non-healing DFUs (Fig 7). On the other hand, there was a reduced abundance of *Arthrobacter* and *Corynebacterium* (Figs 6C and 8, Table 4), which contributed to the decrease in abundance of *Actinobacteria* in non-healing DFUs (Fig 7). However, these changes were not statistically significant.

## Discussion

Microbial profiling of DFUs using 16S rRNA sequencing shows the existence of a heterogeneous community of microorganisms, even in the absence of any overt clinical signs of infection [10, 12, 14, 15]. Several studies have addressed longitudinal changes in the chronic wound microbiome as well as associations between the microbiota and clinical outcomes during the course of treatment, such as debridement and/or antibiotic therapy [16, 17, 26, 27]. However, all these studies addressed bi-weekly or variable treatment regimens. In contrast, weekly treatment’s impact on the wound microbiota is still poorly understood. An understanding of the early temporal changes occurring in microbial composition is important to improve current wound management practices.

In this study, we used 16S rRNA next generation sequencing to examine the compositional changes occurring in the microbiota of DFUs at the patients’ initial visit and one week later to assess the impact of initial standard treatment. Furthermore, we addressed the possibility that the initial DFU microbiome composition could be a prognostic biomarker of healing/non-healing.

Our data showed that the bacterial diversity in DFUs differed between wound and foot skin. Specifically, we showed that DFU wound beds are colonized by a greater number of distinct bacterial phylotypes compared to the wound edge or skin outside the wound at both time points. To our knowledge, this is the first study that showed the difference between microbiomes in these three locations. A previous study that addressed spatial variation of chronic wound microbiota reported minimal differences at different locations only within individual wound beds [40].

Analysis of clinical covariates on DFU microbiota showed significance for sampling location, healing outcome, diabetes type, HbA1C, ABI, Gender and Age. Given the limited number of subjects enrolled in this study, the individual subject microbiota appeared to underlie these confounding clinical variables.

Analyses of the OTUs present in the wound bed samples showed that approximately 80% of the bacterial taxa are the resident microbiota of the foot based on their shared presence in the foot skin outside the wound. The other 20% of the taxa found in the wound bed were commensal microbiota, pathogenic microbiota, and several environmental bacterial species, which is not surprising given that these wounds are exposed to the environment. For example, we detected environmental species of *Arthrobacter*, such as *A*. *cumminsii*, in DFUs. It has been previously shown that *Arthrobacter* species are sometimes present in skin infections [41–43], including DFUs [44–46].

The results of our taxonomic analysis showed that 5 out of 13 phyla were predominantly abundant, making up over 99% of the DFU microbiota. These dominant phyla were *Firmicutes*, *Actinobacteria*, *Proteobacteria*, *Bacteroidetes*, and *Fusobacteria*, consistent with published studies [14, 26, 47]. There were some differences in the proportion of each phylum compared to the published data, which may be attributed to the patient demographics, different sampling techniques, inclusion of control (foot skin outside the wound) and the sequencing targets (V1-V3 vs V4). We found that, out of 142 genera, 12 were predominant in DFUs. While the majority of these predominant genera, notably *Staphylococcus*, *Corynebacterium*, and *Finegoldia*, were in good agreement with the published literature [12, 14, 15, 26, 45, 47], we found a surprisingly low abundance of *Pseudomonas* in our samples. Low abundance of *Pseudomonas* was also observed by Malone *et al*. [47] and may be attributed to differences in patient demographics and environmental factors [12, 15, 45].

A technical limitation in this study prevented the detection of genus *Cutibacterium* (formerly *Propionibacterium*), which is a prominent commensal found on the human skin. Specifically, we were not able to detect *Cutibacterium* because we sequenced the V4 hypervariable region of 16S rRNA [48]. A study by Nelson *et al*. suggested that the absence of *P*. *acnes* in V4 libraries might be attributed to a single nucleotide mismatch in the 16S rRNA gene of *P*. *acnes* that prevents annealing of the 515F primer [49]. Nevertheless, studies of chronic wounds targeting the different 16S rRNA hypervariable regions sensitive for *Cutibacterium* have reported low frequency of occurrence and abundance of this genus in diabetic foot ulcers [12, 15, 16, 45].

Of interest to future studies, we found a substantial presence of *Brevibacterium*, *Oligella*, and *Alcaligenes* that has not been reported previously. Although their contribution to DFU pathogenesis remains unknown, these three bacterial genera have often been implicated in other clinical infections and should be considered in future studies [50–53].

When we examined the impact of initial debridement on DFUs’ microbiota, we found that the bacterial diversities at the first visit and one week follow-up visit were similar. This is in agreement with the studies by Tipton et al. showing that the microbiome communities are more similar within a short time-period [27]. These results also indicate that the DFU microbiota might not have been completely removed by the initial sharp debridement and recolonized the wound within a week, which is consistent with clinical observations that multiple cycles of wound debridement and offloading are necessary to observe even partial wound closures.

When we grouped the wound samples based on the surrogate-healing outcome, we observed that initial abundance of anaerobic bacteria, specifically GPAC (including *Peptoniphilus*, *Anaerococcus*, *Finegoldia*, *and Helcococcus*), in DFU microbiome might be associated with impaired healing. Specifically, we found a statistically significant increase in frequency of occurrence and relative abundance of the genus *Peptoniphilus*. We have also observed increase of *Anaerococcus*, *Finegoldia*, *and Helcococcus*, but this increase was not statistically significant. Based on power analyses, we would need 30 subjects per group to detect any significant difference between healing and non-healing wounds, thus our data should be confirmed on a larger group of subjects.

Sloan et al. also reported the presence of anaerobic bacteria, including Gram-positive anaerobes, in 20 non-healing DFUs and speculated that DFU microbiomes are determined by the ulcer environment and that consistent presence of certain combinations of bacteria may contribute to delayed healing [26].

GPAC are both resident bacteria of the human skin and opportunistic pathogens present in DFUs that are deep, necrotic, and ischemic [54–56]. Studies have shown that GPAC express multiple virulence factors which can trigger inflammation and enhance survival against antibacterial peptides, resulting in increased pathogenicity and impaired wound healing [56]. Although more studies are needed to address this question, it is possible that the abundance of GPAC, especially *Peptoniphilus*, could be used as a predictor of wound healing. Kalan et al. also proposed that the microbiome could serve as a prognostic marker of impaired healing based on the observation that mean proportion of several anaerobic genera such as *Anaerococcus*, *Helcococcus*, *Porphyromonas*, *Prevotella*, and *Veillonella* did not change in unhealed DFUs at 12 weeks post-debridement [17]. In contrast, the same genera were significantly reduced after debridement in DFUs that healed within the same time period. However, the authors did not explain why debridement only affects abundance of anaerobic bacteria in healing wounds. As noted above, what our data shows is that the baseline abundance of several anaerobic genera is higher in non-healing as compared to healing wounds. Perhaps when deep layers of ulcers are in fact colonized by anaerobic bacteria such as GPAC, even sharp debridement could not remove these anaerobes from non-healing ulcers. Additionally, the host response could be impaired and incapable of combating these anaerobes in non-healing DFUs.

We have also observed a decreased abundance of *Arthrobacter* and *Corynebacterium*. A biological explanation as to why a reduction in these genera coincided with impaired DFUs remains unclear at present. It is possible that some of the species belonging to these genera produce antimicrobial agents (such as bacteriocins) that prevent proliferation of pathogenic bacteria, thus having a beneficial role in DFU healing.

## Conclusions

We have observed substantial bacterial diversity and abundance in DFUs that was not significantly altered after one week of standard care. We also found that the bacterial diversity in DFUs differed between wound and foot skin outside the wound at both time points. Our data also revealed an association between a higher abundance of GPAC, especially *Peptoniphilus*, and impaired DFU healing. Thus, abundance of this genus could be used as a predictor of DFU healing.

## Supporting information

S1 Fig Significance and impact of clinical covariates on DFU microbiota. Significance of clinical covariates on DFU wound bed and wound edge microbiota were assessed using PERMANOVA and their impact on beta-diversity using the Bray-Curtis dissimilarity is presented in a NMDS ordination (stress = 0.21). Shown are: (A) Significance of clinical covariates using PERMANOVA, (B) Impacts of individual subjects, (C) blood glucose levels or HbA1c, (D) gender, (E) age, (F) blood flow or Ankle-Brachial Index, (G) and wound duration in months on DFU wound microbiota.
(DOCX)

S1 Table Patients and samples metadata. (XLS)

S2 Table Relative OTU abundance. (XLS)
