# Supplementary material for: Association between baseline abundance of Peptoniphilus, a Gram-positive anaerobic coccus, and wound healing outcomes of DFUs
Source: PLoS One. 2020 Jan 24;15(1):e0227006. doi: 10.1371/journal.pone.0227006 (PMC6980618; doi:10.1371/journal.pone.0227006)

**S1 Fig. Significance and impact of clinical covariates on DFU microbiota**

Significance of clinical covariates on DFU wound bed and wound edge microbiota were assessed using PERMANOVA and their impact on beta-diversity using the Bray-Curtis dissimilarity is presented in a NMDS ordination (stress = 0.21). Shown are: (A) Significance of clinical covariates using PERMANOVA, (B) Impacts of individual subjects, (C) blood glucose levels or HbA1c, (D) gender, (E) age, (F) blood flow or Ankle-Brachial Index, (G) and wound duration in months on DFU wound microbiota.


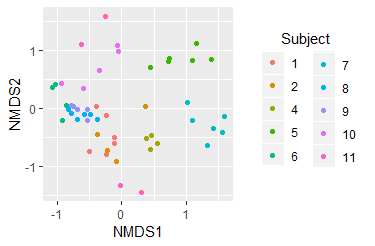

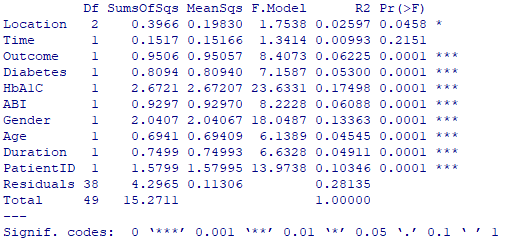
**A B**


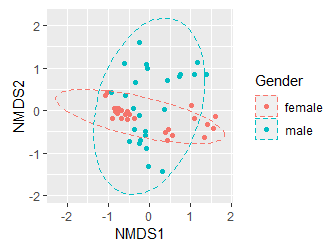


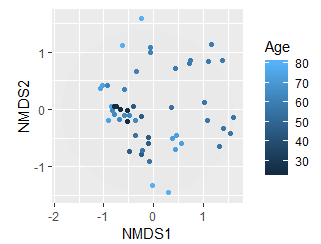

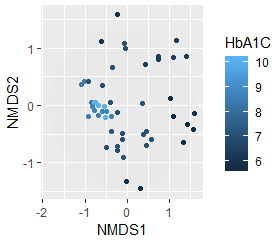
**C D E**


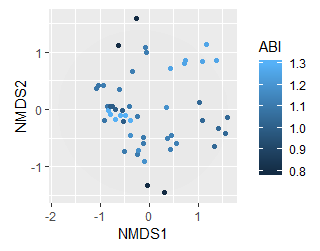


**F G**
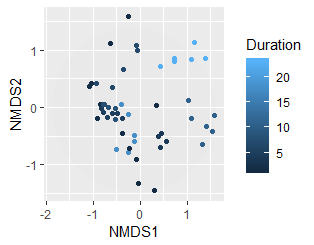

Supplement: S1 Fig — Significance of clinical covariates on DFU wound bed and wound edge microbiota were assessed using PERMANOVA and their impact on beta-diversity using the Bray-Curtis dissimilarity is presented in a NMDS ordination (stress = 0.21). Shown are: (A) Significance of clinical covariates using PERMANOVA, (B) Impacts of individual subjects, (C) blood glucose levels or HbA1c, (D) gender, (E) age, (F) blood flow or Ankle-Brachial Index, (G) and wound duration in months on DFU wound microbiota. (DOCX) [file pone.0227006.s001.docx]
